# Cannabidiol (CBD) Induces Lipid Microdomain Disruption or Budding in Ternary Mixtures

**DOI:** 10.1021/acs.jpcb.5c06965

**Published:** 2026-03-25

**Authors:** C. S. Velez-Saboyá, Francisco A. López-Pérez, Luis G. Rodríguez-Huerta, Francisco J. Sierra-Valdez, J. Roberto Romero-Arias, R. A. Barrio, J. C. Ruiz-Suárez

**Affiliations:** † Centro de Investigación y de Estudios Avanzados-Monterrey, Parque de Investigación e Innovación Tecnológica, Apodaca, Nuevo León 66600, Mexico; ‡ Escuela de Ingeniería y Ciencias, 27746 Tecnológico de Monterrey, Monterrey, Nuevo León 64700, Mexico; § Instituto de Investigaciones en Matemáticas Aplicadas y en Sistemas, U.N.A.M., 01000 CdMx, Mexico; ∥ Instituto de Física, U.N.A.M., 01000 CdMx, Mexico

## Abstract

CBD, one of the main phytocannabinoids present in *Cannabis sativa*, is a singular and promising molecule in medicine, as it is increasingly being used to alleviate diseases derived from inflammation. However, until now, the molecular targets through which it produces its therapeutic effects remain unclear. In fact, since at least 65 physiological targets related to its biological action have been described, it seems that we are far from fully understanding the correct underlying effect. Could it be that the mechanism of action lies elsewhere? To explore the influence of CBD on membranes, we here study its effect on lipid domains in giant and small vesicles. In our experiments, we used fluorescence microscopy, differential scanning calorimetry, isothermal titration calorimetry, and dynamic light scattering to show that in the presence of CBD, lipid domains are disrupted and sometimes lift off. The fission event occurs only near the phase separation temperature, where biological membranes normally function. In addition, we propose a phase field model to theoretically describe the observed budding effect. In the context of recent results showing that degradation of lipid domains can have an impact on analgesia, our findings could provide key elements to consider that the action of CBD on biological cells could be driven by a soft matter phenomenon.

## Graphical abstract

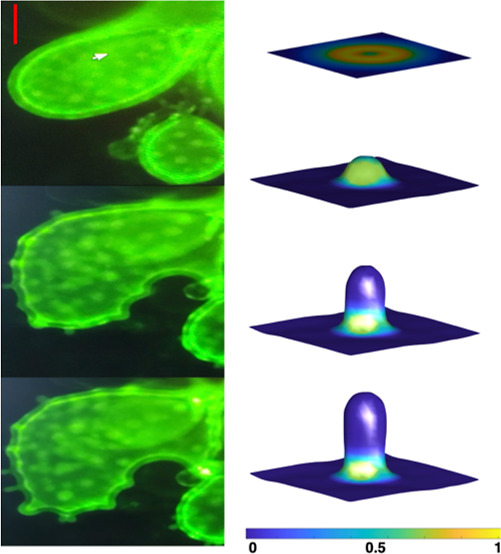

## Introduction

Since the existence of functional lipid domains or rafts in cell membranes was first hypothesized,^1^ solid experimental and numerical evidence has accumulated to accept that they are essential for living cells.^2–6^ The canonical definition states that lipid microdomains are dynamic groups of saturated lipids and cholesterol that move in the fluid membrane and function as platforms for the binding of functional proteins. At physiological temperatures, lipid rafts are found in the ordered liquid phase with characteristics intermediate between the gel and the disordered liquid phase, coexisting with the rest of the membrane, which is in the disordered liquid phase. The high affinity of some proteins for these domains facilitates the formation of complexes and the activation of specific signaling pathways.^3,7^ On the other hand, since lipid rafts participate in the aggregation of misfolded proteins, neuronal damage can occur that leads to neurodegenerative disorders such as Alzheimer’s and Parkinson’s disease.^8–10^

Lipid domains, in model lipid membranes, form when saturated phospholipids and cholesterol attract each other, excluding unsaturated phospholipids.^5^ This exclusion carries a penalty since the system must pay a free energy cost. The origin of this penalty is the concomitant mismatch between the thickness of the lipids in the domain and the excluded lipids outside.^11–13^ Indeed, since the liquid-ordered domains are thicker than those of the liquid-disordered ones, the polar groups of the latter face the hydrophobic groups of the former. This energetically unfavorable situation produces a free energy cost per unit length, called line tension,^11,12^ which controls the domain size, morphology, and vesicle formation in lipid membranes. When line tension is sufficiently large relative to membrane bending rigidity, it favors domain budding, neck constriction, and ultimately vesicle scission. Recent studies^14–16^ demonstrate that line tension is not a fixed material parameter but can be dynamically modulated by membrane composition, temperature, and the presence of small amphipathic molecules, such as serotonin, which alter interfacial packing and phase behavior. Through these physical mechanisms, modulation of line tension provides a protein independent pathway for regulating membrane remodeling, domain stability, and vesiculation in both model membranes and biological contexts. Therefore, the permanent search for free energy reduction labels rafts as dynamic entities, which eventually, by lateral diffusion of their constituents, become larger. At a critical size where the line tension energy term is greater than the Helfrich energy, which describes the elastic energy of a lipid membrane deformation in terms of its geometry by mean curvature, Gaussian curvature and surface tension,^17,18^ the raft can be pushed out of the plane to minimize the contact zone.^11^

If the domains in the actual membranes were not able to lift off, the cells would be fairly inert. In fact, budding is a vital mechanism for biological cells to create cargo vesicles that transport information and molecules between different compartments of the cell.^12^ Budding gives rise to vesicles whose membranes have different properties compared to the donor membrane.^19^ However, budding is a deformation event that requires a high thermal energy (around 100 k_B_T or greater) to spontaneously occur, especially in pure lipid systems. Beyond artificial membranes, for example, in real biological ones where such a source of energy is not available, some agents, such as the endosomal sorting complexes required for transport (ESCRT),^17,20^ or bacterial toxins such as Shiga toxin,^21,22^ help in budding induction.

In this paper, we report new findings regarding the unexpected effect of CBD on lipid microdomains. Indeed, we found that CBD induces the disruption or budding of such lipid domains in giant unilamellar vesicles (GUVs), multilamellar vesicles (MLVs), and small unilamellar vesicles (SUVs). In the first case, we used fluorescence microscopy (FM) to visually observe the perturbed domains; in the second case, we used differential scanning calorimetry (DSC) and isothermal titration calorimetry (ITC) to evaluate their thermodynamic signals produced by the interaction of the molecule; and in the third case, we used dynamic light scattering (DLS) to observe a reduction in size upon raft expelling. Finally, we apply a phase-field model based on a Helfrich curvature model that provides the shape and critical size of the interface determining the lipid rafts, where the difference between the spontaneous curvature of both phases provides the mechanism for budding, and a Gaussian curvature term drives the vesiculation.

It is important to put emphasis on our reasons for this study. First, the budding of lipid domains driven by a hydrophobic molecule is a relevant and novel phenomenon that is important to study in order to advance our understanding of biophysical systems related to membrane instabilities. Despite the fact that the impact of CBD on biological membranes has been reported since about 40 years ago, a long series of multidisciplinary studies have been carried out to dissect the molecular details behind its biological activities.^23^ Computational studies have revealed that CBD can have different locations when it interacts with different membrane composition (POPC or POPE lipids),^24^ and several DSC studies have shown that CBD reduces the melting transition temperature of lipid membranes.^25–27^ Other authors have suggested that CBD and cholesterol may bind to the same site in some proteins, especially those that are localized in cholesterol-enriched domains.^28^

Second, it is of great value to explore a molecule that has a very long history as a therapeutic agent.^29^ Today, medical treatments based on this already approved molecule are exponentially increasing due to the fast evolution from anecdotal to clinical studies in several diseases such as migraine,^30^ diabetes,^31^ epilepsy,^32^ inflammation,^33,34^ among others. These clinical studies have generated a large amount of reports in the literature, excellently reviewed by various authors^29,35^ where many specific CBD targets have been classified. In fact, more than 60 targets, including enzymes, transporters, receptors, and ion channels, have been proposed.^35,36^ Thus, and this gives rise to our third reason: such pharmacological promiscuity,^37–39^ does not allow firm conclusions to be drawn about the mechanism of action of the molecule.

Although it may seem a nontrivial assumption at this point, it is worth exploring the idea that CBD could function as a soft matter phenomenon implicit in biological processes. In other words, if lipid domains (which are soft biomaterials in which even individual lipids diffuse laterally) are crucial platforms for functional proteins, their destabilization produced by CBD could possibly be the origin of the effect. A recent report, which studies the disruption of lipid domains as a possible target of analgesic effects, reinforces our speculation.^40^

## Materials and Methods

### Materials

Lipids 1,2-dioleoyl-*sn*-glycero-3-phosphocholine (DOPC), 1,2-dipalmi- toyl-*sn*-glycero-3-phosphocholine (DPPC), and 1-Myristoyl-2-[12-[(7-nitro-2–1,3-benzoxadiazol-4-yl)­amino]­dodecanoyl]-*sn*-Glycero-3-Phosphocholine (14:0–12:0 NBD PC) were purchased from Avanti Polar Lipids (Alabaster, AL) and used without further purification. Cholesterol (CHO) was purchased from Sigma-Aldrich (St. Louis, MO, USA). Cannabidiol (CBD) (99%) (CAS: 13956-29-1) was purchased in CrescentCanna (New Orleans, USA). Texas Red dye (TR-DHPE) was purchased from Invitrogen. Chloroform, methanol, sucrose, glucose, and dimethyl sulfoxide (DMSO) were purchased from Sigma-Aldrich (Toluca, Mexico). Extran MA 02 was from Merck Mexico (Naucalpan de Juarez, Mexico). Sylgard 184 silicone elastomer base and curing agent were purchased from Dow Corning (Midland, USA). The distilled water was deionized twice with a Milli-Q IQ 7000 Ultrapure Water System from Merck Millipore Mexico (Naucalpan de Juarez, Mexico) before use. Indium tin oxide (ITO) coated coverslips (18 × 18 mm, 100 Ω/sq) were purchased from NANOCS (New York, USA).

### GUVs and Fluorescence Microscopy

GUVs are an invaluable model system in membrane biophysics,^41–45^ used to study a wide set of phenomena, including mimetic cell motility,^46^ lateral molecular diffusion,^47^ shape changes,^48^ and fission effects,^49^ upon the addition of some molecules. To form GUVs, we used the well-known liposome electroformation technique,^50^ recently improved by us.^51^

Two indium tin oxide (ITO) coated square coverslips were cleaned with Extran MA 02 using a cotton swab and rinsed with deionized water. The ITO-coated side of each coverslip was further swabbed with methanol and chloroform before a final water rinse. Each clean ITO-coated Coverlip was attached to the center of a round (42 mm diameter) bare glass Coverlip using polydimethylsiloxane (Sylgard 184) and left to cross-link at 80 °C for 60 min. Finally, a thin strip of copper foil tape was attached to the conductive side of the prepared glass electrodes. An electroformation chamber was assembled using a commercially available device suitable for microscopic examination (POC-R2, Pecon). We used the outer frame of this chamber to hold the two facing electrodes with the help of a screw ring and a silicone gasket (1 mm thick) as a spacer. See further details in a previous report by our group.^51^

To model a lipid raft system, we used a saturated lipid (DPPC) and an unsaturated lipid (DOPC) together with CHO. In the case of fluorescence experiments, we may incorporate TR-DHPE and NBD PC into the lipid mixture before hydration.

The above lipid suspension was prepared to obtain a concentration of 0.5 mg/mL (0.73 mM). It was doped with 1 mol % of NBD-PE 16:0, which goes into the ordered liquid phase, and on some occasions with 1 mol % Texas Red DHPE which prefers the disordered liquid phase. The suspension was divided into two parts: one was reserved as a control and the other was doped with a 5 mol % of CBD. One drop of the control sample and two drops of the other volume were placed on the surface of the ITO. It is worth noting that the aforementioned CBD concentration was selected, among two others with higher concentrations, because it produced the best GUVs; see Figure S4 in the Supporting Information. Before assembly of the chamber, the replicas of the lipid film of each group were hydrated with 20 μL drops of 50 mM sucrose solution preheated to 60 °C. After the electroforming chamber was assembled, a function generator was connected to the copper tape. Thereafter, an alternating current with a sine wave setting was applied at a frequency of 10 Hz and voltage of 1 mV for 60 min. The entire electroformation protocol was performed at a sample temperature of 45 °C using an upper stage incubation system (Incubator PM S1, Insert P S1, Pecon) coupled to the inverted microscope. Most of the budding experiments were performed with the resulting grown lipid membranes, but if on some occasions we needed to separate them from the ITO surface, the alternating current was modified to a frequency of 3 Hz and a voltage of 2 V for half an hour. The electroforming chamber was then cooled to 30 °C and the GUVs were detached from the substrate with gentle manual tapping. After detachment, the drops of each sample were kept separated, the chamber was disassembled, and the sucrose drops were carefully transferred to another ITO. To each drop, we added 30 μL of a 50 mM glucose solution that caused the sedimentation of sucrose-filled GUVs, because it is a little less dense than sucrose, but not too much to cause an osmotic shock. Dual-channel fluorescence photomicrographs (NBD/Texas Red or NBD only) were acquired for vesicle characterization. Image processing was performed with ZEN 2 Pro imaging software.

### Multilamellar Vesicles (MLVs) Preparation

Individual lipids were dissolved in chloroform to obtain a homogeneous mixture of DPPC/DOPC/CHO (0.4:0.4:0.2), for a total lipid concentration of 0.73 mM. The solvent was removed with a constant stream of N_2_ for 40 min and 55 °C to completely dry it and obtain a lipid film. Subsequently, the lipid films were hydrated with HEPES (pH 7.4) at 55 °C, vortexed for 5 s and incubated at 55 °C at 550 rpm for 40 min to lead a suspension of MLVs. The same procedure was used for both the DSC and ITC experiments.

### Differential Scanning Calorimetry (DSC)

The heat capacity profiles of MLV suspensions were recorded by a nanocalorimeter (NanoDSC, TA Instruments) at a constant scan rate of 1 °C/min and constant pressure of 3 atm. Before starting the calorimetric scan, the samples were equilibrated for 5 min at 80 °C and cooling scans were performed from 80 to −10 °C. DSC experiments were performed only twice as a result of the high reproducibility. Thermograms were analyzed with the NanoAnalyze software (v3.12.0; TA Instruments) provided with the instrument. Experiments were carried out with and without the use of 6.1% v/v of DMSO, both in the reference and sample cell. For experiments in which CBD was included, a stock solution of CBD was prepared in DMSO and a corresponding volume fraction was added to an aliquot of MLVs to achieve 5 mol % of CBD and 6.1% v/v of DMSO concentrations, as used in fluorescence experiments. In the following subsection, an explanation is provided as to why this high concentration of DMSO is used.

### Isothermal Titration Calorimetry (ITC)

Calorimetric measurements were performed by titration of a DMSO/CBD complex into the suspension of MLVs using an Affinity ITC (TA Instruments, Newcastle, DE, USA). CBD was previously dissolved in DMSO at a final concentration of 60 mg/mL (190.8 mM). This DMSO/CBD stock is further resuspended in aqueous buffer (HEPES, pH 7.4) to obtain a final concentration of 6.1% v/v of DMSO and 11.63 mM of CBD to finally be used in the syringe as a titrant. The sample cell was conditioned before loading it with a volume of 350 μL of lipid mixtures (0.73 mM) with DMSO in a 6.1% v/v mixture to match the concentration of DMSO in the syringe. Duplicate experiments were performed by titration of 30 2.5 μL injections, with an interval of 600 s between them at 125 rpm. To account for the heat of the dilution, we used the signal corresponding to the titration of the buffer into the buffer for all experiments. To evaluate different thermodynamic states of the membrane system (with and without domain formation), the titration experiment was carried out at 30 and 10 °C, respectively. Data were processed using a Matlab algorithm to calculate the heat of interaction corresponding to each injection and subsequently plot these areas versus the CBD/lipid molar ratio.

We must note that in cell culture experiments, DMSO is regularly used at concentrations lower than 0.1% to maintain suitable cell growth and function, preventing any possible alteration both in lipid and protein receptors at the plasma membrane. Since the main purpose of the ITC experiment was exploring a wide range of concentrations (dose–response curve), the maximum solubility of CBD in DMSO and its required concentration in the titration syringe were such that the experiment was only possible at 6.1% v/v of DMSO to achieve a saturation effect on endothermic peaks. To demonstrate the DMSO effect at such concentration in our ternary lipid system, see Figure S3, which shows only a slight impact on cooperativity in the DSC thermograms.

### Dynamic Light Scattering (DLS)

Dynamic light scattering measurements were performed with a Nano ZSP, Malvern Instruments, United Kingdom, to determine the size distribution of the vesicles at two different temperatures. The laser wavelength and detector angle location were 633 nm and 173°, respectively. Intensity fluctuations were recorded and analyzed using the Stokes–Einstein equation *R* = *K*_B_ *T*/6πη*D*, with *R*, *K*_B_, *T*, η and *D* being the hydrodynamic radius, Boltzmann constant, temperature, dynamic viscosity and diffusion coefficient, respectively. All measurements were performed at 48 and 30 °C, and each measurement was repeated at least three times.

### Theoretical Model

To physically understand our experimental findings (yet to be described), we developed a model to reproduce the processes that occur in our experiments. Our main assumption is that when CBD is incorporated into the membrane, it changes its mean spontaneous curvature locally. Lipid microdomains exist in the membrane primarily due to cholesterol, and CBD molecules preferentially partition into them, resulting in an accumulation of these molecules in such closed regions. The consequence of this process is that a bud is formed because of the spontaneous curvature of the domain. This mechanism continues until enough CBD molecules diffuse within the domain, determining the size and shape of the bud. The bud becomes spherical, forming a region of negative curvature or a neck between the membrane and the bud.

In order to theoretically deal with this system, we have to take into account all the mechanisms that are triggered by the change of local mean spontaneous curvature. We propose adapting a phase field model that has previously been used to study the bending force exerted on a membrane and the topological changes seen in the mitosis of bacteria,^52,53^ phylotaxia,^54^ and vesiculation of a flat membrane caused by thermal fluctuations.^55^ The purpose of our model is to show that CBD produces deformations or protrusions in regions where the membrane has lipid domains.

The model is divided into two parts. The first one accounts for the growth of a closed region on the surface (raft), within which the CBD molecules are being accumulated. The growth of this surface is determined by the distribution of CBD on it. Second, the evolution of the surface is described by a dynamical equation obtained from a free energy functional, whereas the distribution of the CBD is governed by a second dynamical equation obtained from the same energy density. This dynamical evolution causes changes in the spontaneous curvature to reduce the bending energy. A bud is formed, and this needs mass aggregation to account for the excess area and volume of the bud.

Phase-field models have been used to solve interface problems, in which the boundary conditions for the interface follow a conserved order parameter (the phase field) that defines two stable phases.^53,56–61^ In the Ginzburg–Landau approach, two domains are considered that take constant values (typically +1 and −1) and are connected by a diffuse interface of width ϵ, in which the phase field ϕ gradually changes from one phase to the other. Here, ϕ represents the inner and outer sides of the membrane and the interface, where the membrane is usually located at the lotus where ϕ = 0. The local concentration of CBD in the domain is considered a second field *u*. Both fields are conserved quantities, and the dynamic equations are obtained by performing the functional derivatives of the total free energy, namely

1 $$\begin{array}{ll} \frac{\partial \phi }{\partial t}= & \nabla \cdot \left(D_{\phi }\nabla \frac{\delta F}{\delta \phi }\right),\\ \frac{\partial u}{\partial t}= & \nabla \cdot \left(D_{u}\nabla \frac{\delta F}{\delta u}\right). \end{array}$$

where *D*_ϕ_ and *D*_u_ are the corresponding diffusion coefficients, providing the time scales for the system. The total free energy of the vesicle system, F , is given by

2 $$F=\int_{V}\left(A_{b}F_{b}+A_{k}F_{K}+A_{s}V_{s}+A_{f}V_{f}+\sigma |\nabla \phi {|}^{2}\right)dV$$

where the contributions are

(i) Considering the Ginzburg–Landau formalism, the bending and spontaneous curvature term $F_{b}$ is written as,^54,55^

3 $$F_{b}={\left(\left(\phi -\epsilon C_{0}\right)\left(\phi^{2}-1\right)-\epsilon^{2}\nabla^{2}\phi \right)}^{2}$$

where *C*_0_ is the term of spontaneous curvature that describes the natural tendency of the vesicle to acquire a shape with a certain nonzero spontaneous curvature. Since spontaneous curvature is locally modified by the local concentration of CBD, we follow the approach of Barrio et al.^53^ and consider the term spontaneous curvature as a function of $u:C_{0}=C_{0}\left(u\right)=\beta {\left(u-u_{0}\right)}^{2}$ , where β measures the strength of the interaction, that is, the ability of CBD to modify spontaneous curvature and *u*_0_ is a threshold value. *A*_*b*_ is the bending modulus.^55^

(ii) The formation of a protrusion requires a topological change of the membrane surface, as stated by the Gauss-Bonnet theorem. This is accomplished by considering the energetics of the Gaussian curvature, measured by the Gaussian modulus, *A*_*k*_, in eq 2. The Gaussian curvature contribution $F_{K}$

4 $$F_{K}=\sum_{i<j}\frac{{\left(1-\phi^{2}\right)}^{2}}{2}\left(Q_{ii}Q_{jj}-Q_{ij}^{2}\right)$$

where *Q*_*ij*_ is the curvature tensor^54,55^

(iii) We now consider modeling the dynamical behavior of the order parameter *u*. We assume that the potential energy felt by the system is different near the interface (*V*_s_) than far from it (*V*_f_). We consider that *V*_s_ is very small far from the interface, therefore proportional to (ϕ – 1) and *V*_f_ is only noticeable far from it and proportional to ϕ. Then, the terms in eq 2 corresponding to *V*_s_ and *V*_f_ are associated with the interaction between the membrane and CBD:

5 $$\begin{array}{ll} V_{s} & ={\left(\phi^{2}-1\right)}^{2}{\left(u-u_{\min}\right)}^{2}{\left(u-u_{\max}\right)}^{2}+\lambda |\nabla u{|}^{2},\\ V_{f} & =\phi^{2}{\left(u-u_{far}\right)}^{2} \end{array}$$

where *u*_min_, *u*_max_ represent the affinity of the CBD molecules to attach near the cholesterol molecules, and *u*_far_ is the stable concentration of CBD far from the interface. In the present system they are taken to be *u*_min_ = *u*_far_ = 0 and *u*_max_ = 1, which simulate the adhesion of CDB to the interface. The second term is a surface tension energy that minimizes the area of the raft, that is, λ|∇*u*|^2^ allows the CBD concentration to diffuse into the membrane while minimizing the area of the boundary where CBD is present. The parameter σ is a Lagrange multiplayer that conserves the area of the membrane.

In order to account for the preference of CBD molecules to attach to the lipid domain, we need to increase the amount of material around the membrane. Thus, we introduce an additional term that adds mass to the domain.

We propose that the arrival of CBD to the lipid domain could be modeled by means of a normal distribution *G*[*u*] of CBD centered on the axis of the lipid raft and near the interface. The center of this 3D function follows the displacement of the summit for a certain time τ, until there is no more CBD in the system to join the rat. Therefore, the dynamical system looks like this

6 $$\begin{array}{l} \frac{\partial \phi }{\partial t}=D_{\phi }\nabla^{2}\left(\frac{\delta F}{\delta \phi }\right)+mG\left[u\right]\Theta \left(t-\tau \right)\\ \frac{\partial u}{\partial t}=D_{u}\nabla^{2}\left(\frac{\delta F}{\delta u}\right)+G\left[u\right] \end{array}$$

where Θ is a Heaviside function. The volume and area of the membrane increase as mass is added, represented by *m* > 0.

Finally, the explicit expressions of the dynamical equations in terms of ϕ and *u* are the following

7 $$\begin{array}{l} \frac{\partial \phi }{\partial t}=D_{\phi }\nabla^{2}(A_{b}(2\mu \left[\phi \right]\left(3\phi^{2}-1-2\epsilon \phi \beta {\left(u-u_{0}\right)}^{2}\right)\\ -2\epsilon^{2}\nabla^{2}\mu \left[\phi \right])+4\phi A_{s}\left(\phi^{2}-1\right){\left(u-u_{\min}\right)}^{2}{\left(u-u_{\max}\right)}^{2}\\ \begin{array}{l} +2\phi A_{f}{\left(u-u_{far}\right)}^{2}-2\sigma \nabla^{2}\phi \\ -A_{K}\left(\frac{12\phi }{1-\phi^{2}}T_{1}+\frac{2\left(3\phi^{2}+1\right)}{{\left(1-\phi^{2}\right)}^{2}}T_{2}\right))+mG\left[u\right]\Theta \left(t-\tau \right)\\ \begin{array}{l} \frac{\partial u}{\partial t}=D_{u}\nabla^{2}\{4\epsilon A_{b}\beta u\left(1-\phi^{2}\right)\mu \left[\phi \right]+2A_{s}[\left(u-u_{\min}\right)\times \\ \left(u-u_{\max}\right)\left(2u-\left(u_{\min}+u_{\max}\right)\right){\left(\phi^{2}-1\right)}^{2}-\lambda \nabla^{2}u]\\ +2\phi^{2}A_{f}\left(u-u_{far}\right)\}+G\left[u\right] \end{array} \end{array} \end{array}$$

where

8 $$\begin{array}{l} \mu \left[\phi \right]=\left(\phi -\epsilon \beta {\left(u-u_{0}\right)}^{2}\right)\left(\phi^{2}-1\right)-\epsilon^{2}\nabla^{2}\phi \\ T_{1}=\phi_{xx}\phi_{yy}+\phi_{xx}\phi_{zz}+\phi_{yy}\phi_{zz}-{\left(\phi_{xy}\right)}^{2}-{\left(\phi_{xz}\right)}^{2}\\ \begin{array}{l} -{\left(\phi_{yz}\right)}^{2}\\ T_{2}=\phi_{xx}{\left(\phi_{y}\right)}^{2}+\phi_{xx}{\left(\phi_{z}\right)}^{2}+\phi_{yy}{\left(\phi_{x}\right)}^{2}+\phi_{yy}{\left(\phi_{z}\right)}^{2}\\ \begin{array}{l} +\phi_{zz}{\left(\phi_{x}\right)}^{2}+\phi_{zz}{\left(\phi_{y}\right)}^{2}-2\phi_{x}\phi_{y}\phi_{xy}-2\phi_{x}\phi_{z}\phi_{xz}\\ -2\phi_{y}\phi_{z}\phi_{yz} \end{array} \end{array} \end{array}$$

and ϕ_*x*_*i*__ = ∂ϕ/∂*x*_*i*_.

## Results

### Experimental Section

In Figure 1 we show a representative fluorescent GUV with lipid domains (green) and the structures of the three lipids used in their formation, CBD and dyes.

**Figure 1:**
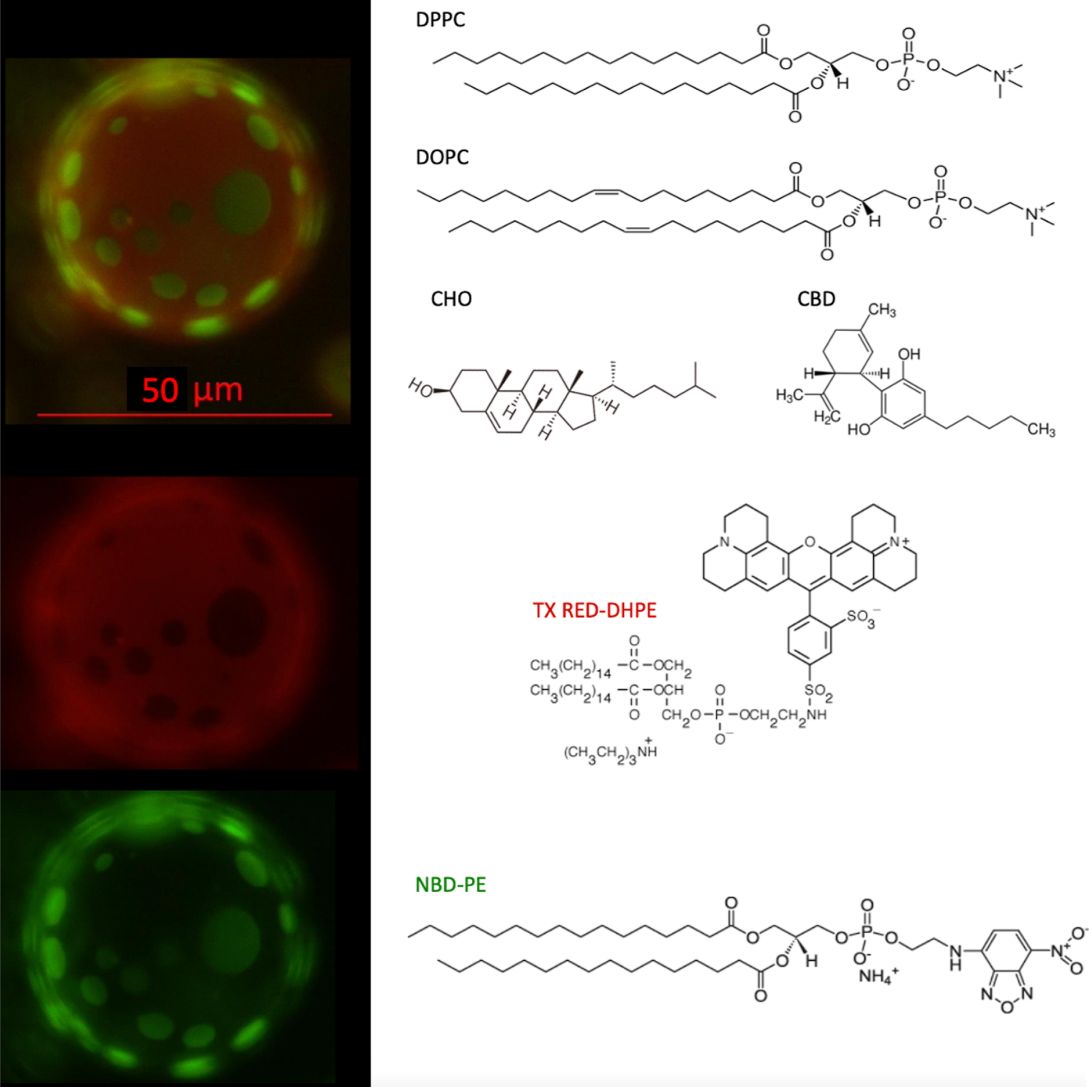
Left panel (top) shows GUVs with fluorescence lipid domains (green zones) immersed in the disordered liquid phase (red zone). Left panel (bottom) shows GUVs with the red and green dyes individually. Right panel shows the structures of the six molecules used in this work.

Figure 2 shows fluorescence micrographs of GUVs, filled with sucrose and precipitated in a glucose solution (see panel A). The temperature of the experiments was slightly below the phase separation temperature (30 °C).^62,63^

**Figure 2:**
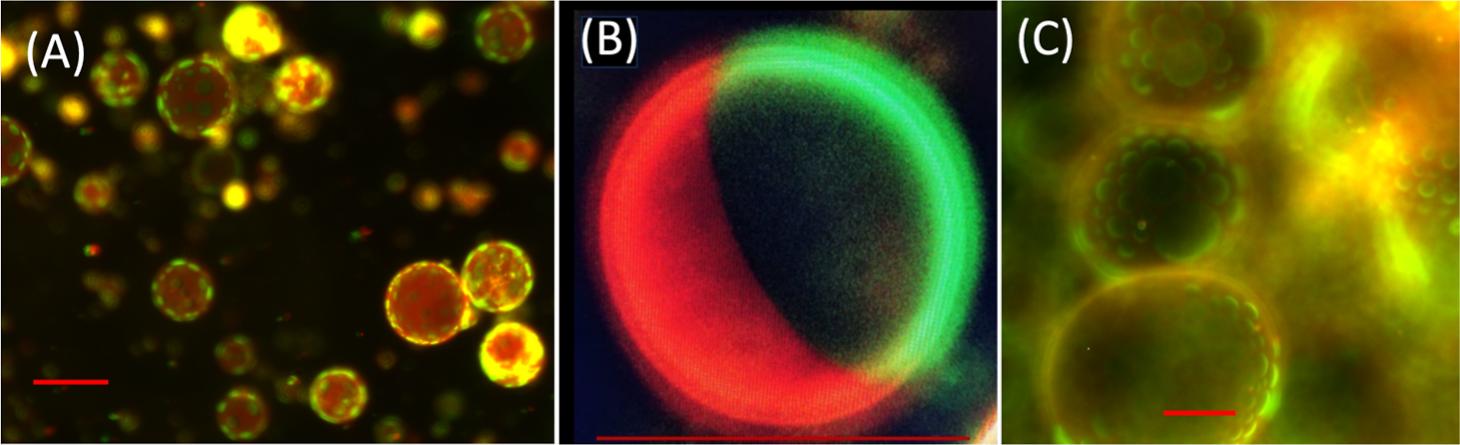
(A) Fluorescence micrograph of GUVs of a lipid mixture of 0.73 mM (DPPC/DOPC/CHO/CBD) with a zero CBD concentration (0.4:0.4:0.2:0.0). Note that the lipid domains (green zones) are flat, see also Figure 3A. (B) The result of complete domain coalescence (Janus particle), phenomenon occurring after several hours or even days in the absence of CBD (many of these Janus particles can also be observed in Figure S1A). (C) The effect produced by CBD with a concentration of 5 mol % (0.38:0.38:0.19:0.05). Lipid domains are not flat, but noticeably protruding. Bar scale is 50 μm. The experiments were performed at 30 °C.

In the absence of CBD, lipid domains coalesce into Janus particles after several hours or even days (Figures 2B and S1A), while the buds slowly develop in the presence of CBD, see the protrusions in Figures 2C and S1. It is important to note that it is difficult to follow the growth of the buds because the giant vesicles move and rotate, so focus is lost. Hence, snapshots such as the one shown in Figures 2C and S1 are only indicative of the dynamics of the initiation process. The full budding dynamics can be clearly observed if the GUVs are not detached from the ITO surface (see Materials and Methods) because their movement is hindered. A sequence of photographs that shows the dynamics of the budding effect is depicted in Figure 3B, compared to a control case without CBD (A) (see also Movies S1, S2, and S3). Furthermore, it is worth highlighting that not only is it more difficult to film the budding dynamics in detached spherical GUVs, but, as we will see later, budding processes occur in such a case with much more difficulty (from the energetic point of view) than in nondetached GUVs. To evaluate the importance of cholesterol, we also proved that in mixtures where there is no cholesterol, the lipid domains are not formed; see Figure S2.

**Figure 3:**
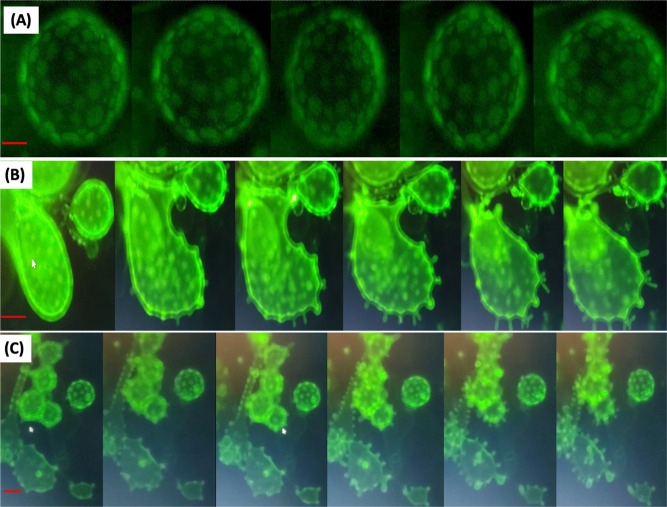
Fluorescence micrographs of three different samples (ternary mixtures of DPPC/DOPC/CHO) (0.73 mM). (A) Lipid membrane of a nondetached GUV with rafts in the absence of CBD. The sequence does not show budding. (B) Sequence of lipid membranes in nondetached GUVs where budding of microdomains is observed. CBD (5 mol %) was incorporated since the beginning (together with the lipid mixture before electroformation). (C) Sequence of lipid membranes in nondetached GUVs where budding of microdomains is observed. CBD was first dissolved in flax oil and then incorporated to the GUVs through a nanoemulsion at a concentration of 1% v/v. In all cases the sequences go from 0 to 60 s (see also Movie S3 in the Supporting Information). The temperature was 30 °C. The length of the red bars is 12 μm. Note that to obtain the time-lapse of the budding, only one fluorescent lipid is used. We performed a statistical analysis of budding produced by CBD; see Figures S5A and S6.

Now that we have observed through fluorescence microscopy the budding or disruption of lipid domains in GUVs, our next goal is to reduce their size and look for such processes at the nanoscale. Not only is the mere existence of nanometric-sized lipid rafts a topic of great interest, but the observation of their possible budding from lipid membranes will be especially relevant. In fact, the dynamics of the lipid domains at the nanoscale plays a crucial role in real cells.

However, because of their size, we cannot see them with an optical microscope as we did with GUVs. To assess this impossibility, we performed a DLS experiment using unilamellar vesicles; We clearly observed the effect that CBD has on the production of lipid raft budding; see Figure 4: Vesicles’ size reduces after budding.

**Figure 4:**
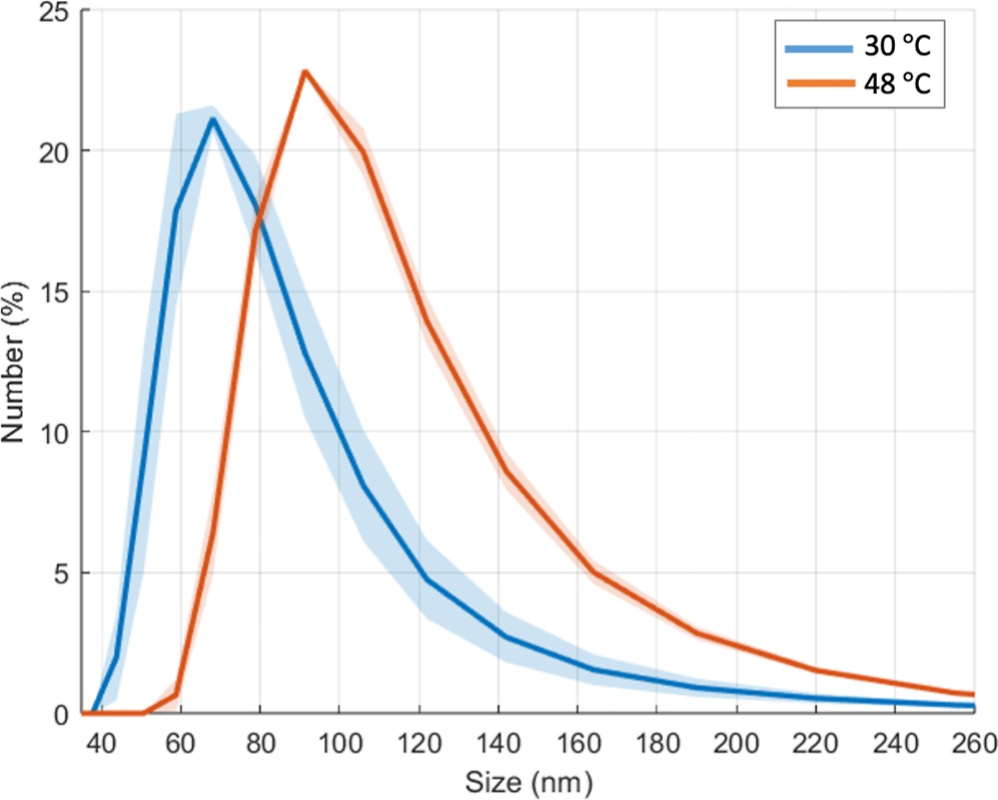
Size distributions of small unilamellar vesicles (SUVs) with the lipid ternary mixture of 0.73 mM and 5 mol % of CBD, at two different temperatures: 48 and 30 °C. At 48 °C there are no microdomains (the lipids are homogeneously mixed), at 30 °C, lower than 35 °C at which domains form (see Figure S5), it is expected the domains lift off. Indeed, the shift to the left (blue line) indicates that budding takes place, reducing the size of the SUVs.

Furthermore, to inquire in more detail about the molecular interactions between CBD and lipid membranes from an energetic perspective, we implemented calorimetric strategies. It is important to mention that the only calorimetric study performed to date in these particular ternary mixtures has demonstrated a gel-to-fluid phase transition or melting temperature (*T*_m_, in the temperature range of −20 to −15 °C).^64^ Using a highly sensitive calorimeter, our results show, for the first time, a phase-separation phenomenon that occurs approximately between 10 and 40 °C (see Figure 5A). Regarding this issue, ternary mixtures of high- (DPPC) and low- (DOPC) chain melting temperature lipids, in addition to cholesterol, have been reported to undergo lateral phase separation into two coexisting liquid phases (liquid-ordered, *L*_o_ and liquid-disordered, *L*_α_) at a temperature well-known as critical temperature or *T*_c_.^65^ There is the possibility, however, that at 10 °C, a three-phase region (*l*_*d*_ + *l*_*o*_ + *g*) would form under our mixture conditions,^66^ which would not affect our results.

**Figure 5:**
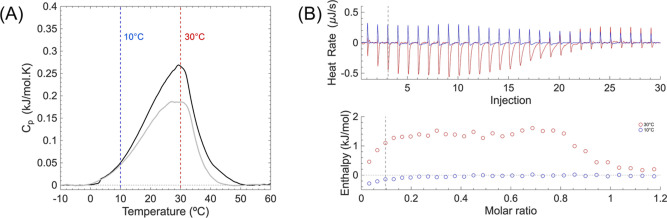
(A) Representative DSC thermogram of (DPPC/DOPC/CHO) (0.73 mM) liposomes showing the phase separation transition with an average *T*_m_ = 30.3 °C ± 1.22 °C and Δ*H* = 5.36 kJ/mol ± 0.43 kJ/mol (black trace). The effect of 5 mol % of CBD shows a *T*_m_ = 27.8 °C ± 1.56 °C and Δ*H* = 3.93 kJ/mol ± 0.38 kJ/mol (gray trace). (B) Representative isothermal calorimetric titrations of CBD on DPPC/DOPC/CHO liposomes, at 10 and 30 °C, and enthalpies for both cases. Blue peaks indicate exothermic signals, red ones indicate endothermic signals. Third injection (dashed line) approximately corresponds to the accumulated 5 mol % of CBD in DMSO, also illustrated in (A).

Below *T*_c_, there is not enough thermal energy (*k*_B_ *T*) to disrupt the *L*_o_ phase. The domains are circular, have smooth edges, and after some time collide and coalesce to produce a Janus particle, as observed in Figure 2B. As the temperature increases toward *T*_c_, the edges of the domain become rough and small, since the membrane now absorbs energy in the form of heat from the system to produce growth. *T*_c_ has been denoted as the point where there is no significant density difference between the phases *L*_o_ and *L*_α_, and therefore an observable phase separation occurs, as commonly observed in various fluorescence microscopy experiments (see also Figures 2 and 3). This thermal cutoff point (*T*_c_) is also known as the phase separation temperature.^65^ Beyond *T*_c_, no domains remain; instead, compositional fluctuations appear and disappear over time. Indeed, it has been demonstrated in similar ternary mixtures by Honerkamp-Smith et al. that the domain line tension reaches their minimum value precisely between 30 and 31 °C.^67^ In particular, it can be observed that *T*_c_ corresponds to the temperature found in our thermogram with the maximum *C*_p_ value (see Figure 5). Therefore, we speculate that such a transition corresponds to the energetic process behind phase separation, where the area under the curve can be approximated to the enthalpy change required to disrupt the domain.

To investigate such energetic events, we performed ITC experiments in which CBD is titrated into a DPPC/DOPC/CHO liposome suspension. To achieve this, we performed the experiments under two different conditions: at 10 °C, where probably a Janus particle is already formed, and at 30 °C, where coexistence of liquid-ordered/liquid-disordered phases or rafts exist. Our results show that CBD titration at 10 °C induces exothermic signals, characteristic of its partitioning in the middle of the membrane. However, when CBD titration is performed at 30 °C, there is clear evidence of negative heat peaks, indicating a possible melting phenomenon due to weakening of cohesion within cholesterol-rich domains; see Figure 5A. This, in turn, may promote an unfavorable energetic state or surface tension, which ultimately results in protrusions or budding. An enthalpy plot is also shown for both temperatures (Figure 5B). From this particular experiment, system saturation can be observed after 25 injections.

### Theoretical

As mentioned in the Materials and Methods section, our goal was to mathematically estimate the effect that CBD had on the stability of the lipid domains. It is therefore worth noting first that the partitioning of CBD into rafts is driven by hydrophobic forces, which reduce the free energy when CBD interacts with the membrane, for example, with cholesterol. According to Israelachvili and Pashley,^68^ the change in free energy in this case is Δ*G* ≈ −84*R* kJ/mol, where *R* = *R*_1_ *R*_2_/(*R*_1_ + *R*_2_), being *R*_1_ and *R*_2_ the radii of CBD and cholesterol, respectively. Using the reported molar volumes (MV) for both molecules: 306.6 and 391.4 cm^3^, respectively (see ChemSpider Home Page http://www.chemspider.com/), we get (solving for *R* in 4/3 π *R* ^3^ = MV/NA, where NA is the Avogadro number) 0.495 and 0.537 nm. Therefore, we find Δ*G* ≈ −21.6 kJ/mol. It is important to clarify that CBD must also interact with the other two lipids (DPPC and DOPC). In fact, it is easy to show that the Δ*G*s are similar to the one we calculated for cholesterol.

The model given above (eqs 6) gives us the dynamics of the lipid domain once CBD joins it. Such eq 6 are highly nonlinear so we must solve them numerically. The Euler forward method has already been shown to be reliable in the integration of this system, provided the time step Δ*t* is small enough.^54,55^

We work with a grid of size Δ*x* between the points, which is constant along the system and equal throughout the 3D domain. The scales chosen for the implementation were Δ*x* = 1 for space and Δ*t* = 10^–4^ for time. We impose zero-flux boundary conditions on the boundaries of the domain. The initial conditions for the domain are a planar shape with a small perturbation in the center of the shape. Simulations are performed in a 3D domain grid with parameters *Nx* = *Ny* = 22, *Nz* = 18. The surface tension coefficients were fixed to σ = 0.1, λ = 0.1. Bud formation requires −*A*_K_ < 2*A*_b_, while vesicle formation requires a topological transition where −*A*_K_ > 2*A*_b_ as was theoretically demonstrated by the phase diagram of Figure 4 in ref 55. For the first case, we took *A*_b_ = 1 and *A*_K_ = 0.5 (see Figure 6) and for the second case we chose *A*_b_ = 1 and *A*_K_ = −10, see Figure S7.

**Figure 6:**
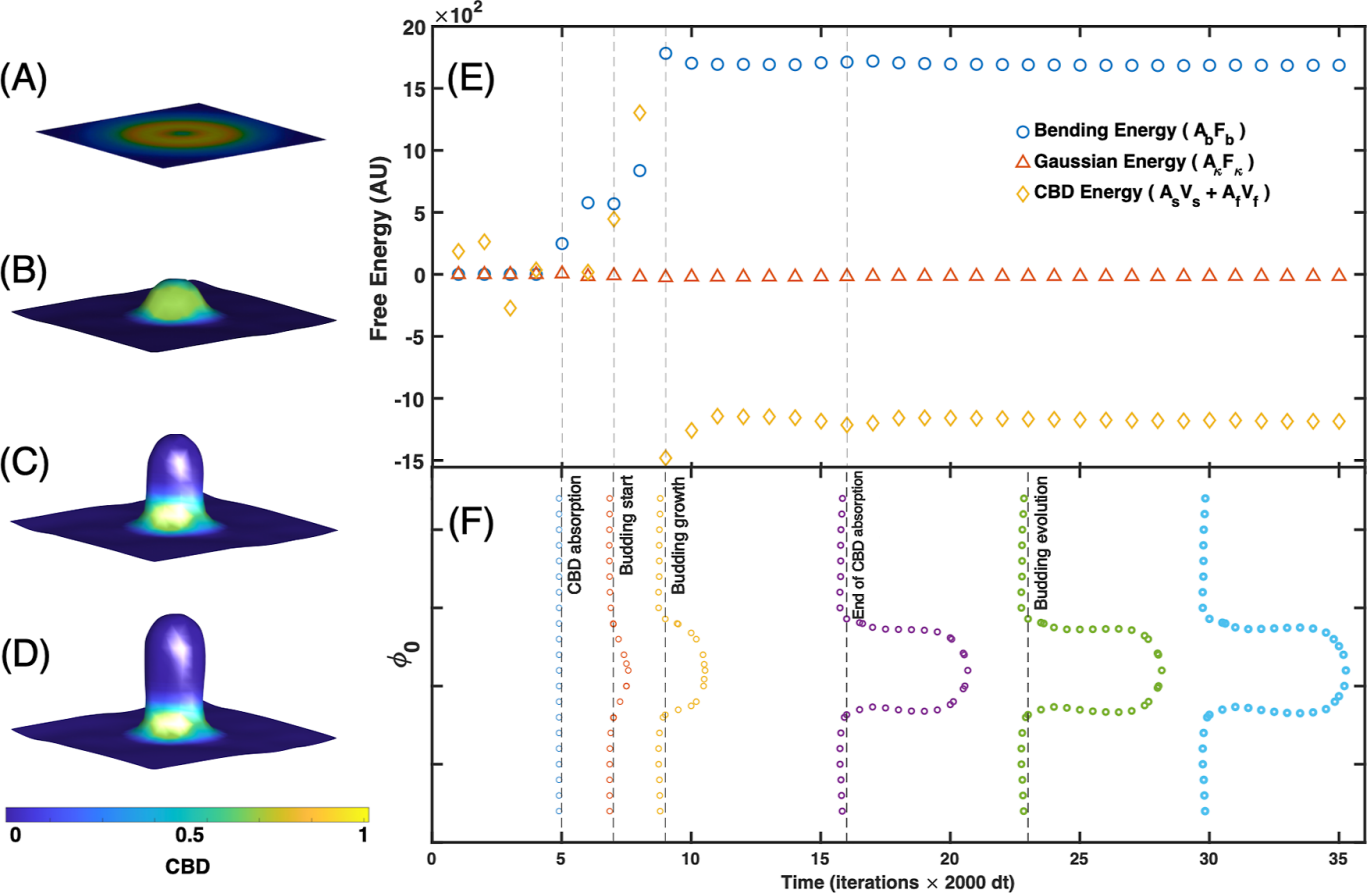
Numerical calculation of the dynamical evolution of an interface. (A) The initial time when the system is a flat surface. The color code corresponds to *u* (CBD concentration) obeying a Gaussian distribution centered on the middle of the square domain. (B) Formation of a bulge due to the variation of the local curvature with CBD concentration. (C) As time progresses, the local curvature of the bulge dominates locally and a neck is formed. (D) At even larger times, the bud develops with a greater neck. (E) Free energy distribution of eq 2 during CBD absorption, budding initiation and neck formation. We use dimensionless units, AU. (F) Time evolution of ϕ_0_ profile.

The other parameters take the following values: *D*_ϕ_ = 2, *D*_u_ = 0.1, ϵ = 1, *A*_s_ = 2, *A*_f_ = 2, β = −0.05, *u*_min_ = 0, *u*_max_ = 1, *u*_far_ = 0, *u*_0_ = 2, *m* = 6 and τ = 10^4^ *dt*. The Gaussian distribution follows the expression $G\left[u\right]=\frac{u_{0}}{2}e^{\left[{\left(x-N_{x}/2\right)}^{2}+{\left(y-N_{y}/2\right)}^{2}+{\left(z-H_{z}\right)}^{2}\right]}/2\gamma$ with *H*_*z*_ varying with growth, following the distance of the source to the uppermost point of ϕ_0_. The width of this Gaussian, γ = 4.8, is related to the size of the raft, and it turns out to be important for the formation of vesicles. In the Appendix, we analyze the parametric stabilization of CBD absorption and vesicle formation on a membrane, using linear theory and estimating the dispersion relation of small perturbations around the flat interface. We observe that local binding of CBD at the interface is crucial to reduce free energy and then produce a bud and a vesicle.

In Figure 6, we show the numerical evolution of an interface during the initiation of the bud and the formation of the neck. It is important to note that our calculation considers a flat membrane, which is the less favorable case for budding. In a spherical membrane such as the one studied here, budding would be much easier, since spontaneous curvature favors the formation of buds. In Figure 6A, the interface starts initially (*t* = 0) as a flat surface, while the concentration of CBD (represented by field *u*) follows a Gaussian distribution centered in the middle of the square domain. Due to variations in local curvature and interactions with CBD concentration, a bulge emerges at the interface (see Figure 6B). As time progresses, the increasing local curvature of the bulge leads to the formation of a neck (see Figure 6C,D). Throughout this process, the evolution of each term to the free energy in eq 2 is depicted in Figure 6E. Note that CBD energy is reduced while the bud is forming due to the local curvature change; see Figure 6F.

The energy contributions associated with Gaussian curvature can promote the transformation of the bud into a spherical vesicle, which eventually detaches from the main interface (see Figure S7).

## Discussion

We have shown by fluorescence microscopy that CBD drives the budding of lipid domains in GUVs. We found that this phenomenon occurs with greater preference in nondetached GUVs (GUVs that are not released from the ITO surface). The reason why this happens is simple: the global energy in detached GUVs is minimal, so there is no further free energy gain in the growth of the bud. In contrast, when GUVs are still bound to the ITO surface, the global energy is not minimal, so budding helps to decrease it.

Before continuing, we mention that some experiments were carried out using other hydrophobic molecules: olive oil and β-Caryophyllene. Olive oil is primarily composed of linoleic, stearic, and palmitic acids. These are as hydrophobic as CBD; indeed, their partition coefficients are, respectively: 7.18, 8.22, and 7.15 (for CBD, LogP is 7.03), see https://www.chemspider.com/search. β-Caryophyllene, which has a partition coefficient of 6.78, is considered a dietary cannabinoid.^69^ In Figures S5, and S6 we show that olive oil and β-Caryophyllene produced much less budding compared to CBD, 10 min after the domains formed. It should be noted that the temperatures at which the lipid rafts appear are different for each case: 35 °C for CBD, 38 °C for olive oil and 34 °C for β-Caryophyllene. Considering that the stearic and palmitic acids of olive oil are saturated, their linear forms may favor ordering, so the ordered liquids of the rafts appear at higher temperatures.

Furthermore, it is important to mention that CBD and similar hydrophobic molecules dissolve indistinguishably between both leaflets within several dozen nanoseconds after entering the membrane from one side. These findings were previously published by our group, using molecular dynamic calculations together with calorimetric experiments and similar molar lipid-to-drug ratios.^26^ Therefore, our fluorescent microscopy experiments reveal that lipid budding phenomena occur regardless of whether CBD is previously incorporated into the lipid membrane (Figure 3B) or added externally once vesicles are formed (Figure 3C). Since lipid budding occurs within seconds in titration experiments, we suggest that this process arises from a homogeneous drug partitioning in both membrane leaflets.

Reducing the size of liposomal entities is important because then we would reach the size of real cells. However, because cells are of micrometric size, we would have to pay the price of not being able to see small areas such as lipid domains at the nanometric scale. To avoid this problem, we take advantage of the fact that a structural modification or budding is an endothermic process. Therefore, we performed thermodynamic experiments with ITC to energetically analyze such phenomena in much smaller liposomes. We clearly observed negative heat peaks representative of endothermic responses when CBD come into contact and perturb them, specifically at the temperature at which phase separation is observed in fluorescence microscopy experiments. It is important to mention that other authors have reported similar ITC experiments by titrating nonpolar drugs (ibuprofen, Naproxen, Diclofenac) into liposome systems that are quite far from their phase transition temperature, thus observing exothermic responses. However, no endothermic peaks have been reported so far regarding drug binding and action in membrane systems.^70^ In addition, it is also interesting how endothermic peaks start to increase while membranes accumulate CBD. In fact, they last for a certain time and eventually the membrane becomes saturated to a point where there are probably no more cholesterol-enriched domains to excrete (see Figure S7B).

An important point to mention before continuing is that the CBD titration process used in ITC experiments is difficult to implement in our microscopic measurements. The reason is that when CBD is delivered to the GUV sample examined under the microscope (via DMSO or other carriers such as a nanoemulsion), the light scattering produced by the colloidal carriers is so high that the fluorescence signal is blocked. However, we succeeded in doing the delivery of CBD by reducing the concentration of the carriers (which in this case a nanoemulsion gave better results). In Figure 3C we show a sequence of images that describe the dynamics of the budding growth after adding CBD (as a nanoemulsion) to the GUVs. See also Movie S3. This visual result may support the endothermic signals (red peaks) obtained in Figure 5B.

The CBD-driven budding phenomenon in lipid domains, which to our knowledge has not been observed before, is interesting from a membrane biophysics perspective. However, we consider that the main impact of our findings is in the direction of a possible correlation of budding driven by CBD action with real-life functions. In fact, we wonder whether the development of such a lipid microdomain modification, produced by CBD, can be a plausible mechanism to explain its effect on neurons.

To venture a well-founded speculation on this issue, we recall that lipid rafts have been proposed to play an important role around plasma proteins. For example: the nicotinic acetylcholine channel, opioid receptors (OR), P2X purinoreceptor 3 (P2X3), neurokinin 1 receptors (NK1R), Toll-like receptors (TLR) and TRP channels;^40,71–80^ the emblematic receptors for endocannabinoides are CB_1_ and CB_2_ receptors, which are seven transmembrane domain G-proteins that form the endocannabinoid system in the central nervous system (CNS).^81–83^ At least one of them, CB_1R_, is believed to be associated or localized in the lipid domains.^84^ Furthermore, in previous works some of us proved that a general anesthetic produced a disruption of neuronal lipid domains with the concomitant dissociation of NMDA and GABA receptors from these domains,^85^ Kashnik et al. recently reported the entrapment of ibuprofen-SL molecules by lipid domains,^86^ and Nehr-Majoros et al. found that a disruption of lipid rafts may affect protein receptors and thus offer novel therapeutic approaches that differ from classical pharmacological receptor antagonism.^40^

In summary, it can be concluded that if a channel or receptor anchored in lipid microdomains results in a modulated biochemical mechanism, it is plausible to think that a lipid-modifying drug, such as CBD, would produce an effect through budding action. In other words, beyond the report of Nehr-Majoros et al., who proposed the modification of cholesterol or a saturated lipid in the liquid-ordered domains,^40^ why not produce a change in mechanical stability using a molecule like CBD? If this were the case, a budding or domain alteration mechanism could be a unifying principle or a missing link that would give meaning to the observed multitarget action of the molecule. Although we found that CBD produces a raft-budding phenomenon in model membranes, our findings could open the door to real cell studies in this direction. As noted briefly before, cell growth of lipid compartments is a strategy to deal with energy imbalances between line tension and curvature-dependent energies in their membranes,^17^ driven mainly by proteins, lipids or the adsorption of small or large biomolecules.^13^ In fact, if proteins themselves induce curvatures in biological membranes,^87,88^ smaller molecules could synergistically trigger budding upon adsorption.^13^

## Conclusions

We have experimentally demonstrated, and theoretically described by a phase field model, that CBD induces the protrusion, modification, or budding of lipid domains in lipid membranes. The reason for these effects is that CBD modifies the spontaneous curvature of the lipid domains in such a way that there is a reduction in bending energy. In this work, we propose that to unravel the mystery that arises with the large number of targets reported so far in the literature on the action of CBD, it is perhaps important to consider the particular effect that this molecule induces on the lipid domains where the targets are anchored. CBD has an undoubted effect on the nervous system and it would be fascinating if the raft-modification mechanism behind its action were feasible. We hope that this report can open the door to future research needed to improve our understanding of this phenomenon. Finally, since minor budding effects were observed with other hydrophobic molecules, it would be interesting to conduct similar studies for these or other molecules.

## Supplementary Material





## Figures and Tables

**Figure 7:**
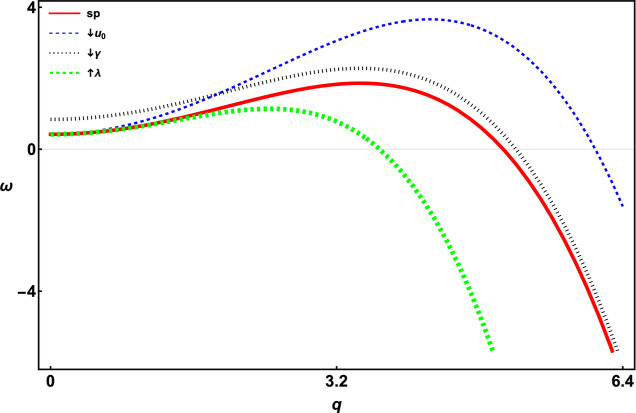
Dispersion relation for different values of the system parameters (sp) as compared to the curve obtained with parameter values (red line) used in Figure 6. Blue dashed and black dotted lines correspond to a decreases of *u*_0_ and γ, respectively and independently, while in the green dotted line the value of λ is increasing.

## Appendix

Here, we analyze the parametric stabilization of CBD absorption and budding formation on a membrane. We study the effects of small perturbations around the flat interface of the form $\mathrm{\phi ̅}={\mathrm{\phi ̅}}_{0}e^{iq\cdot x-\omega t}$ , near ϕ̅ ≈ 0, with small amplitudes of ${\mathrm{\phi ̅}}_{0}≔\left(\phi ,u\right)$ . Here q = *q*_α_ and x = *x*_α_ are the wave vector and Cartesian coordinates. Substituting these expressions into the dynamical equations (eq 1) and considering isotropic perturbations as in refs.,^18,52,55^ one finds that the dispersion relation takes the form

9
$$\omega \left(q\right)=D_{u}\left[4A_{b}\epsilon^{2}\beta u_{0}^{2}+2A_{s}\left(2u_{\min}u_{\max}+{\left(u_{\min}+u_{\max}\right)}^{2}\right)\right]q^{2}-D_{u}\lambda q^{4}+\frac{2x_{0}}{\gamma }G\left[u_{0}\right]$$

and *G*[*u*_0_] is the linearization of the initial Gaussian distribution of CBD at the position *x*_0_.

In Figure 7, we show the dispersion relation eq 9 using the values of the parameters used in Figure 6 in red. When the parameter γ is increased from 2.4 to 4.8, (black dotted line), we notice that there are no major changes, either in the amplitude of the instability or the maximum of the curve (critical length). This is reasonable because the width of the Gaussian distribution is not crucial to starting the instability, provided that it is large enough. Observe that the critical length diminishes when *u*_0_ changes from 1 to 2 (blue dashed line). This parameter regulates the value of spontaneous curvature in the membrane, and doubling its value means that the local curvature of the places where CBD accumulates is greater and the raft formed is smaller (larger critical *q*). The parameter λ measures the energy needed to create a surface where *u* is attached. When its value changes from 0.1 to 0.2 (dotted green line), it exhibits an increase in the critical length, as expected, since it will be more difficult to excite the instability.

## Notes

Movies S1, S2 and S3 (ZIP)
Fluorescence micrographs of different samples. Fluorescence micrograph of electroformed lipid membranes of DPPC/DOPC (with 5 mol% of CBD) (PDF)
