# Supplementary material for: Cannabidiol (CBD) Induces Lipid Microdomain Disruption or Budding in Ternary Mixtures
Source: J Phys Chem B. 2026 Mar 25;130(14):3960–72. doi: 10.1021/acs.jpcb.5c06965 (PMC13071920; doi:10.1021/acs.jpcb.5c06965)
Supplement: Supplementary file 2 [file jp5c06965_si_002.pdf]

Cannabidiol (CBD) induces lipid microdomain disruption or budding in ternary mixtures

Supporting Information

C. S. Velez-Saboyá,† Francisco A. López-Pérez,† Luis G. Rodríguez-Huerta,‡ Francisco J. Sierra-Valdez,‡ J. Roberto Romero-Arias,¶ R. A. Barrio,§ and J. C. Ruiz-Suárez*,†

†*Centro de Investigación y de Estudios Avanzados-Monterrey, Parque de Investigación e Innovación Tecnológica, Apodaca, N.L. 66600, Mexico.*

‡*School of Engineering and Sciences, Tecnológico de Monterrey, Monterrey, N.L. 64849, Mexico.*

¶*Instituto de Investigaciones en Matemáticas Aplicadas y en Sistemas, U.N.A.M., 01000 CdMx, Mexico*

§*Instituto de Física, U.N.A.M., 01000, CdMx, Mexico.*

E-mail: cruiz@cinvestav.mx

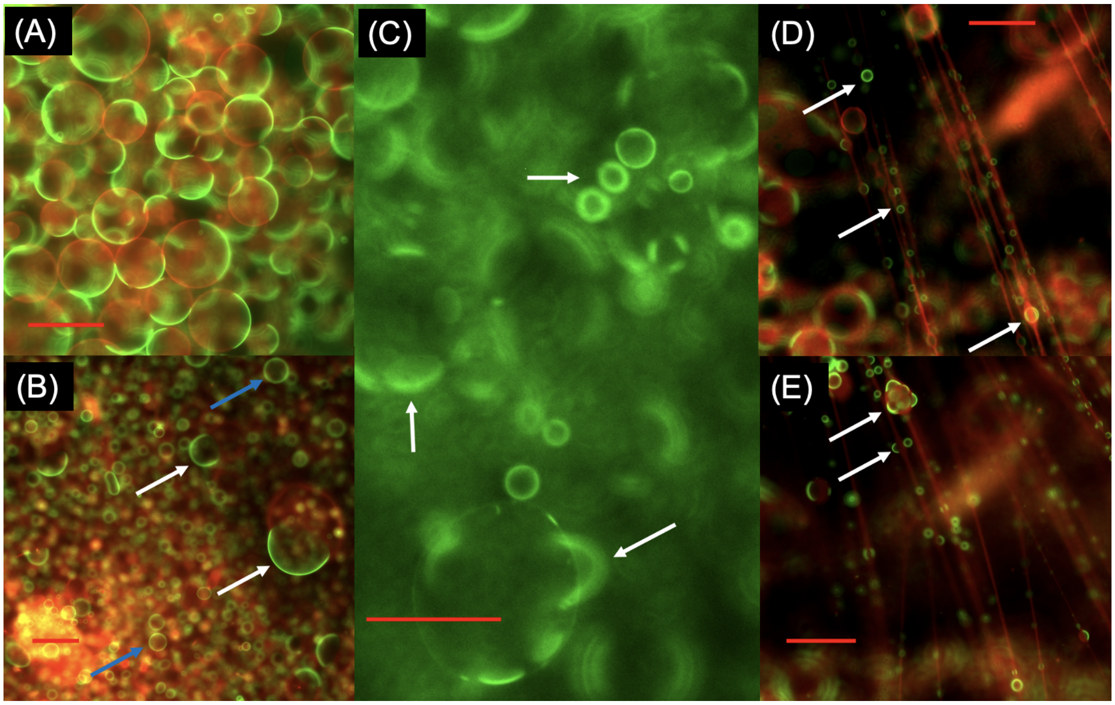

Figure S1: Fluorescence micrographs of different samples. Panel (A) shows Janus particles in the absence of CBD after several hours of incubation at 30 °C. Panel (B) is a sample with 5 mol% of CBD. Note budding events (see white arrows) as well as domains already expelled (blue arrows). In panel (C) these budding events and detached domains are even more evident (white arrows). Panels (D and E) show tubular filaments where budding as well as domains already detached are clearly observed. Red tubular filaments indicate DOPC:Chol disordered liquid phase, while green buds and vesicles indicate DPPC:Chol ordered liquid phase. The length of the red bars is 100 μm.

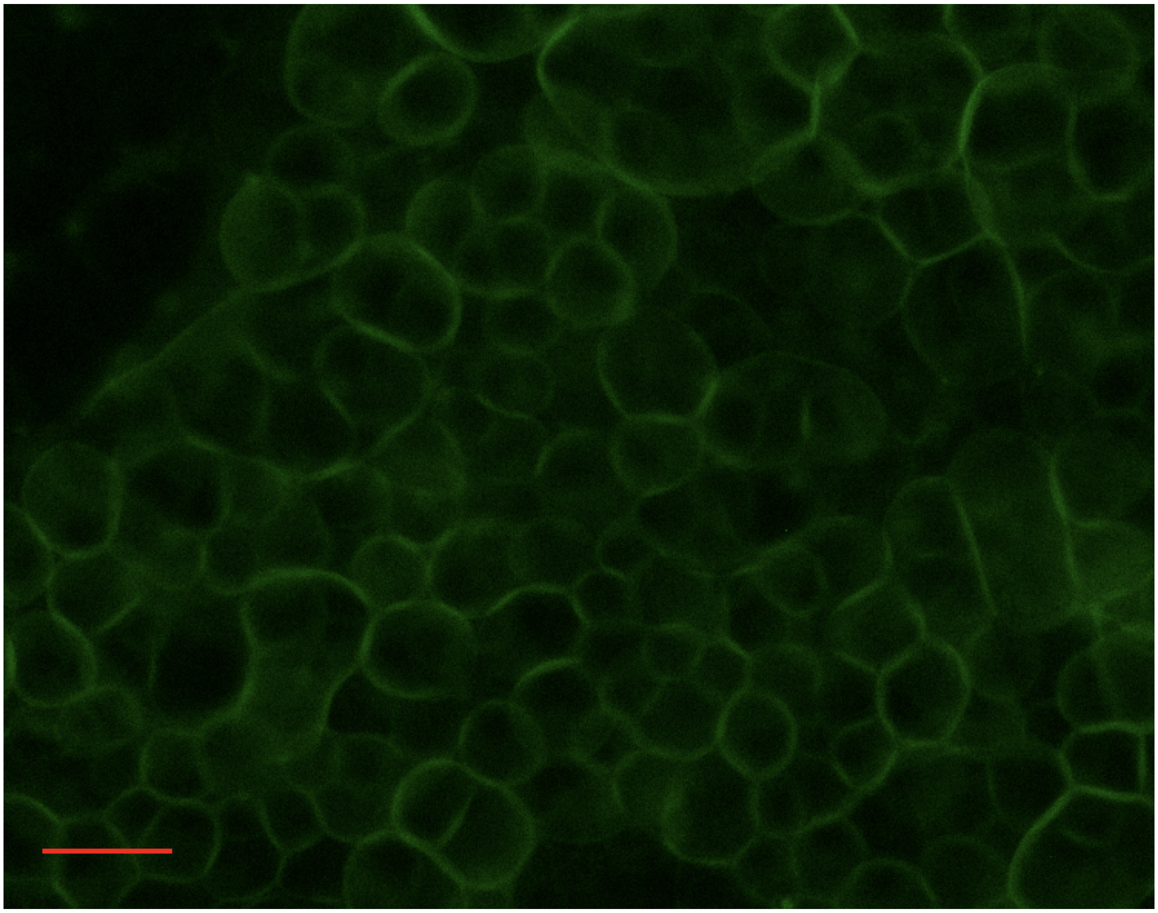

Figure S2: Fluorescence micrograph of electroformed lipid membranes of DPPC:DOPC (with 5 mol% of CBD). In the absence of cholesterol, lipid domains are not formed regardless the temperature. The length of the red bars is 50 μm.

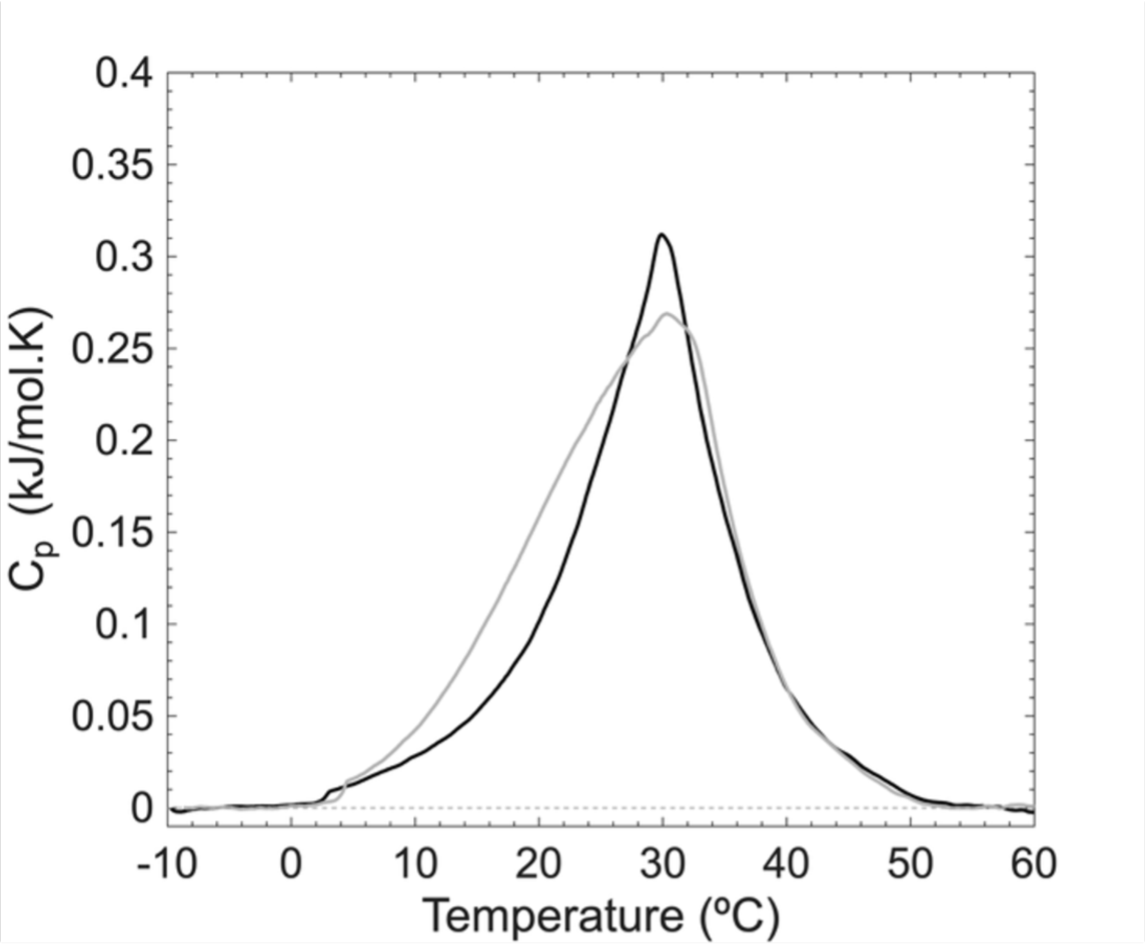

Figure S3: Representative DSC thermogram of DPPC:DOPC:CHO liposomes with (gray trace) and without (black trace) the effect of 6.1% v/v DMSO in the buffer. In DMSO conditions an average T_m = 30.3 °C ± 1.22 °C and ΔH = 5.36 kJ/mol ± 0.43 kJ/mol was obtained, while for non-DMSO conditions a T_m = 29.8 °C ± 1.20 °C and ΔH = 4.72 kJ/mol ± 0.48 kJ/m.

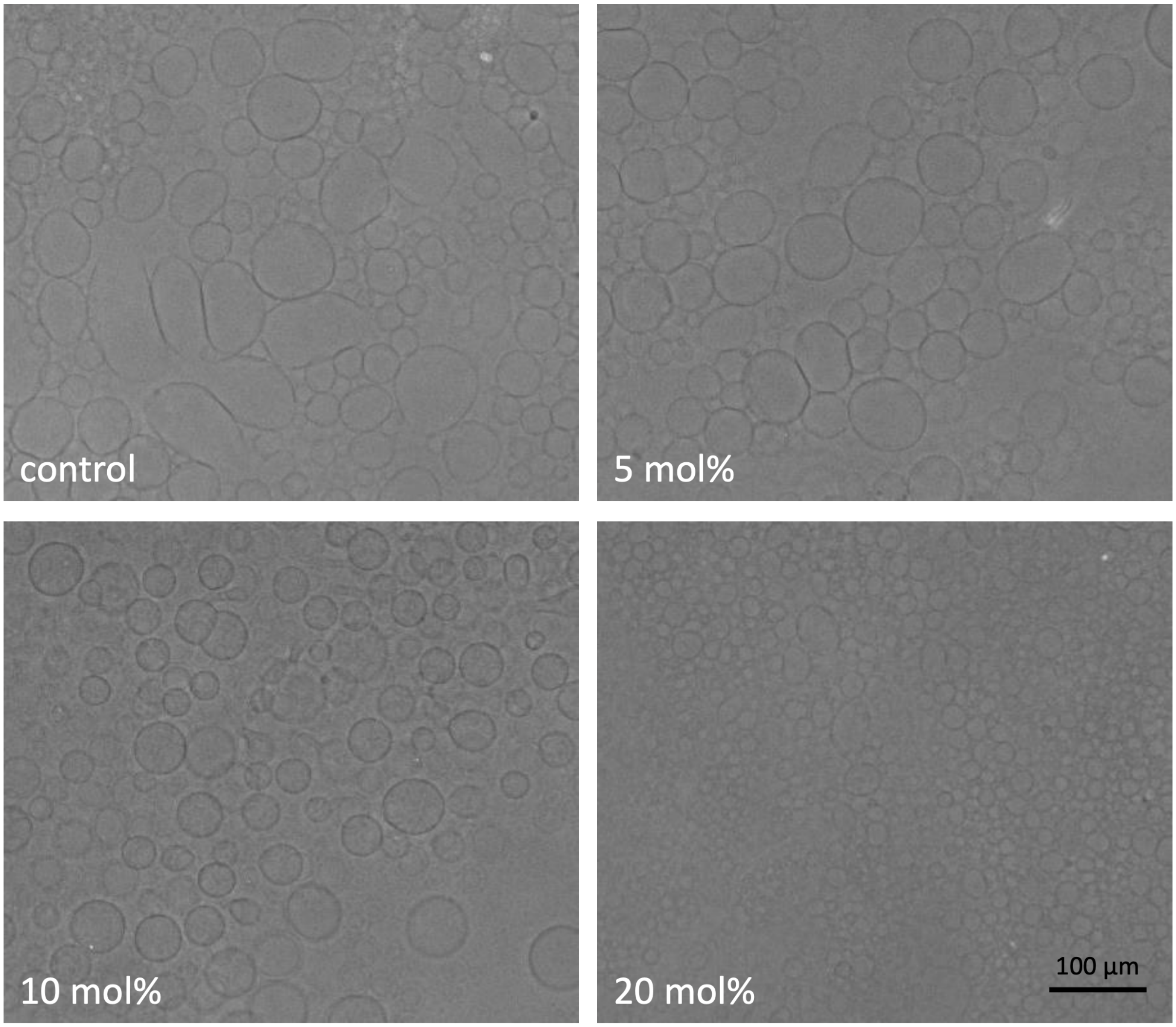

Figure S4: Phase contrast micrographs showing GUVs from the DPPC:DOPC:CHO lipid mixture still adhered to a surface of indium tin oxide (ITO). It is observed that the GUVs resulting from the electroforming process change when adding CBD at 5, 10 and 20 mol%, respectively. It is clear that the best formed and larger GUVs are the ones with less CBD concentration.

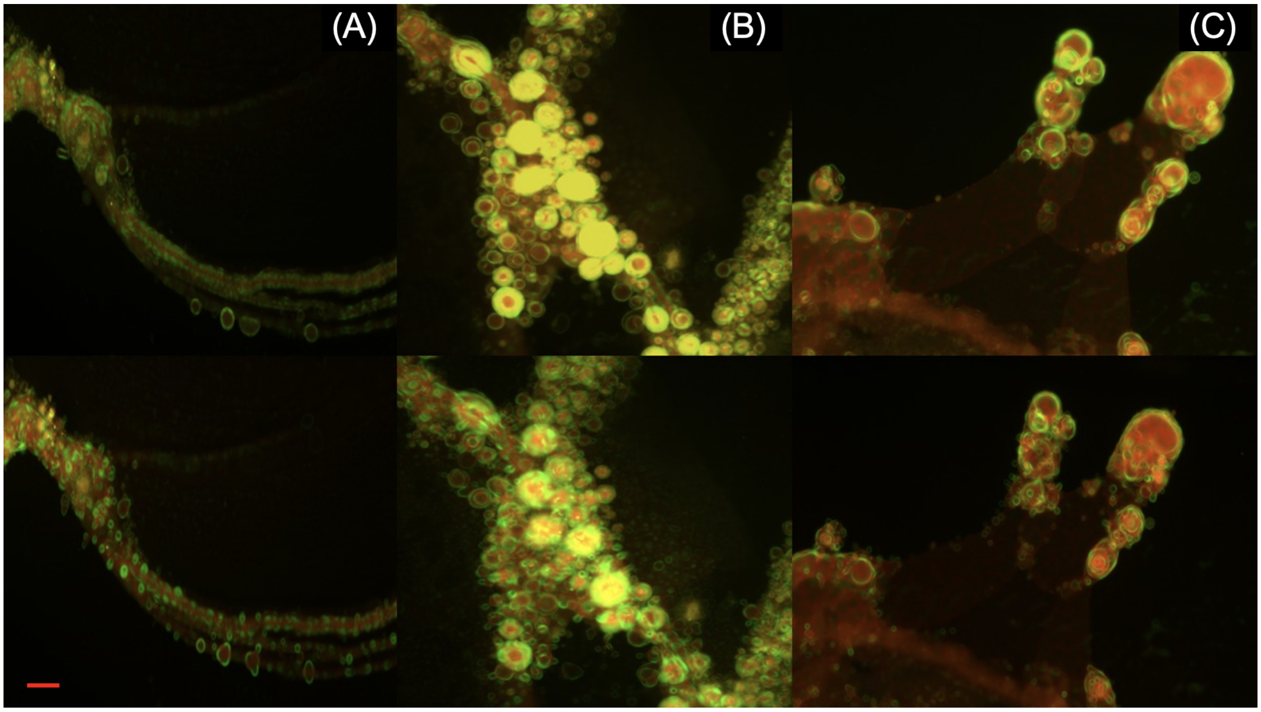

Figure S5: Lipid rafts formed in a membrane made of a DPPC:DOPC:CHOL mixture, with the addition of three different hydrophobic molecules at a 5 mol%: (A) CBD, (B) Olive Oil; (C) β-Caryophyllene. The temperatures at which the domains are formed are: 35 °C, 38 °C, and 34 °C, respectively. The upper panels are within the first minute of formation, the lower panels correspond to 10 minutes after formation. The length of the red bars is 50 μm.

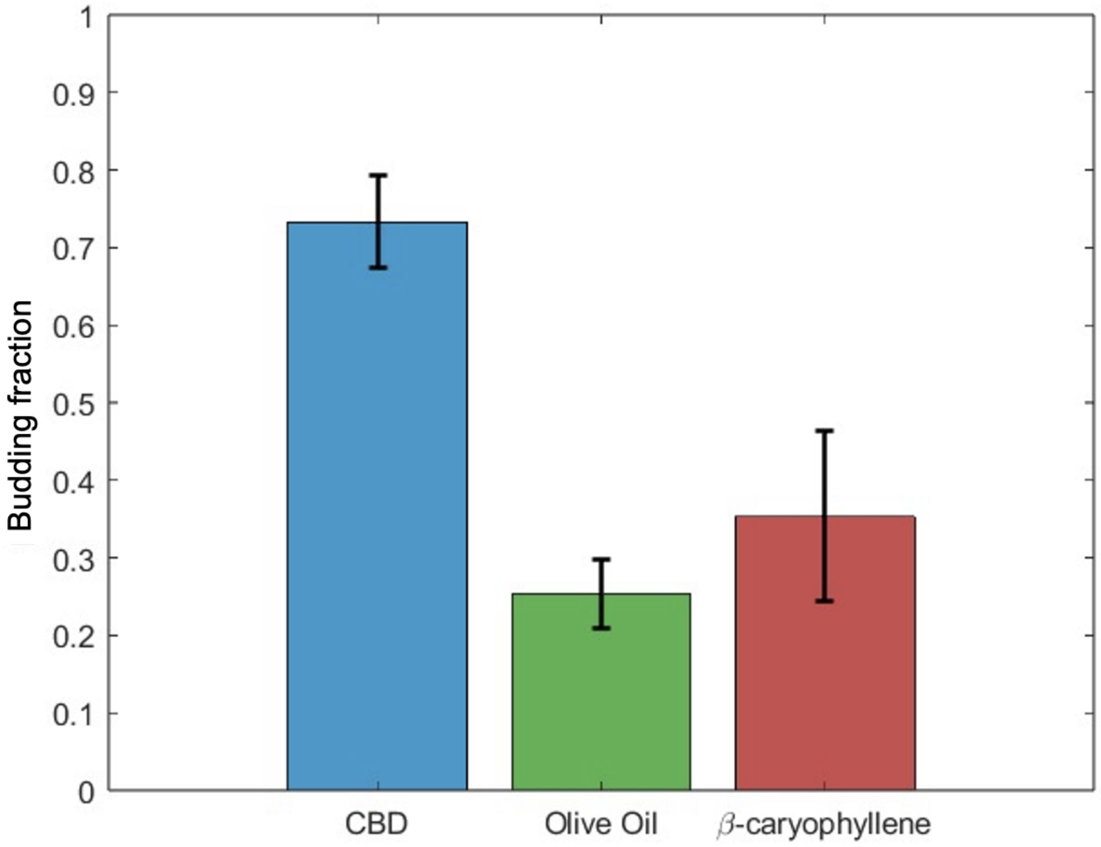

Figure S6: Lipid rafts are counted in three different zones in one experiment, 1 minute after their formation and the averaged is plotted (the images shown in Figure S5 are representative of one zone). Subsequently, the domains exhibiting budding are counted (10 minutes later). We plot the budding fraction (buds/total). The error bars are the standard deviations. The results are reproducible between experiments.

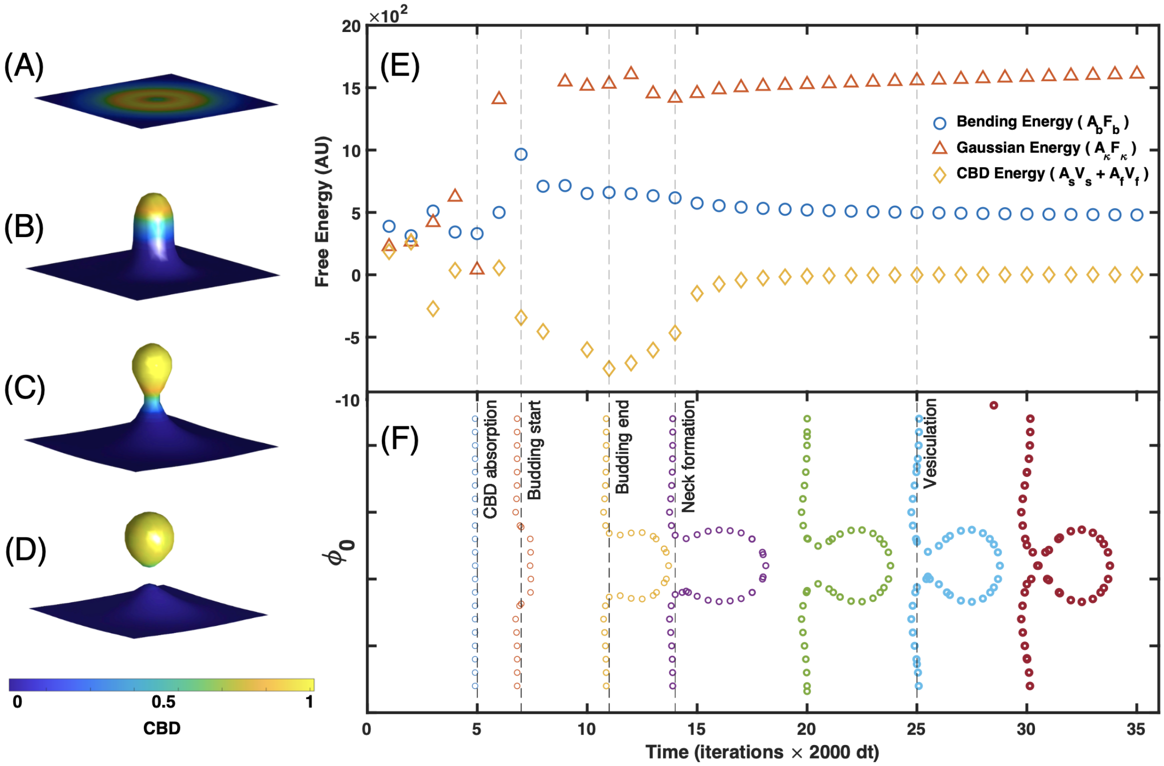

Figure S7: Numerical calculation of the dynamical evolution of a interface. (A) the initial time when the system is a flat surface. The color code corresponds to u (CBD concentration) obeying a Gaussian distribution centered on the middle of the square domain. (B) Formation of a bulge due to the variation of the local curvature with CBD concentration. (C) As time progresses, the local curvature of the bulge dominates locally and a neck is formed. (D) Due to the energy terms related to the Gaussian curvature, a spherical vesicle is formed and detached. (E) Free energy distribution during CBD absorption, budding initiation and vesicle formation. We use adimensional units, AU. (F) Time evolution of ϕ_0 profile.
